# Impact of occupational pairing on women’s fertility plans: roles of domestic burden and housework satisfaction

**DOI:** 10.3389/fsoc.2026.1617449

**Published:** 2026-03-02

**Authors:** Yuan Dang

**Affiliations:** Department of Sociology, Fudan University, Shanghai, China

**Keywords:** fertility intention, GSEM, objective and subjective aspects of housework, occupational assortative marriage, suppression effects

## Abstract

This article examines how the division of housework mediates the relationship between intra-couple socio-economic differences and wives’ fertility intentions.Both objective and subjective aspects of housework equality are considered. Using data from the China Family Panel Studies (CFPS), this study employs generalized structural equation modeling (GSEM) to analyze multiple mediation pathways linking occupational assortative mating to fertility intentions. The results show that mating patterns influence household labor dynamics, with women who “marry up” bearing a heavier housework load. The paradox of contented wives who undertake most housework suggests that (in)equality does not necessarily correlate with (dis)satisfaction. Multiple mediation analysis reveals that both the actual division of housework and wives’ perceived satisfaction with this suppress the direct relationship between occupational assortative mating and wives’ fertility intentions. These two mediators function as multiple suppressors in heterogamous marriages. In non-core middle-class and working-class homogamy, only the suppression effect of wives’ relative housework burden is significant.

## Background

China’s total fertility rate fell below replacement level in the early 1990s, and negative population inertia has since taken hold. While very low fertility and population aging are common across many societies, China’s case is distinctive in the compressed timing of its demographic transition and the legacy of long-term birth control policies, which together constrain the scope for fertility recovery. Understanding the factors influencing the fertility intentions of the childbearing-age population has become a crucial topic in social science. Extant research analyzes the heterogeneities in fertility intentions and actual behaviors between socio-economic groups from the perspective of assortative mating or the (in)equalities in socio-economic resources between spouses (Bagavos, 2017; Krzyżanowska and Mascie-Taylor, 2014; Mascie-Taylor, 1986; Nitsche et al., 2018; Nomes and van Bavel, 2016; Qing, 2024; Sun and Zhao, 2022; Tian et al., 2023; Zhao, 2019). These studies show how women’s fertility is linked to whom they marry in a socio-cultural context, but the mechanisms behind different mating patterns and fertility intentions remain unclear.

To better understand these mechanisms, it is important to examine them within the specific socio-cultural and institutional context of contemporary China, where rapid educational expansion, persistent gender norms, and historical policy legacies shape both partner selection and domestic gender arrangements. On the one hand, rising rates of high-level educational homogamy and hypogamy are driven by women’s greater improvement in education relative to their male counterparts (Cao and Qian, 2024). On the other hand, the domestic division of labor remains highly gendered, with women continuing to shoulder a disproportionate share of housework even in dual-earner households (Chen, 2024; Ji et al., 2017).

The “career-family dilemma” faced by women has been identified in numerous studies (Anderson and Kohler, 2015; Goldin, 2021; Ji and Zheng, 2018; Wilkins, 2019; Yang, 2016) as a major factor in low fertility intentions. McDonald (2000a) explained that lower fertility intentions among women result from the interplay between increased gender equality in individual-oriented institutions (e.g., improvements in women’s education opportunities and labor force participation) and persistent gender inequality in family-oriented institutions (e.g., substantial domestic labor burdens). When career women anticipate conflict between motherhood and career advancement, they are more inclined to prefer smaller families (McDonald, 2000b). Scholars (Esping-Andersen and Billari, 2015; Goldscheider et al., 2015) focusing on the “stalled gender revolution” argue that a husband’s active involvement in household chores could help alleviate women’s “dual-burden” and increase their motivation to have more children. The positive effect of gender equality within households on fertility has been confirmed by numerous empirical studies (Kwan and Choi, 2022; Thomas et al., 2023; Zhao and Ji, 2019).

However, some studies found that women who bear a larger share of housework report higher fertility intentions (Torr and Short, 2004), while others report no significant relationship (Cheng and Hsu, 2020; Yang, 2017). Therefore, focusing solely on the objective household division of labor is insufficient, as it overlooks the intermediate factors that mediate the impact on wives’ fertility intentions (Cheung, 2023). The (in)equality in housework allocation must be “perceived” to influence subsequent decision-making (Neyer et al., 2013).

Scant research has explored the relationships among the actual division of domestic labor, wives’ subjective evaluation of husbands’ participation in housework, and fertility intentions (Cheung, 2023; Riederer et al., 2019; Snopkowski, 2023; Zhang et al., 2023). Cheung’s (2023) research on Hong Kong and Zhang et al.’s (2023) study on mainland China provide empirical support for the mediating role of wives’ satisfaction with housework. Both studies acknowledge the heterogeneity of the association between housework and childbearing across groups and control for socio-economic traits (such as spousal educational level and income). However, they do not incorporate the effects of assortative matching into their analytical framework.

Resource bargaining and social exchange theories suggest that differences in socio-economic status between spouses influence the proportion of domestic labor that the wife is expected to perform (Blood and Wolfe, 1960; Evertsson and Nermo, 2007; Lam et al., 2012; Roman and Vogler, 1999; Stauder and Röhlke, 2022) and affect their subjective evaluation of the fairness of housework distribution (Lennon and Rosenfield, 1994). Although Cheung (2023) and Zhang et al. (2023) suggest using wives’ satisfaction with housework as an intermediate mechanism, they did not validate this causal with rigorous mediation analyses, such as (Generalized) Structural Equation Modeling (GSEM).

Therefore, this study uses the GSEM model to analyze how the division of housework and subjective feelings toward it mediate and explain the effect of assortative matching on wives’ fertility intentions. Differences in socio-economic status between spouses are measured using the Erikson-Goldthorpe-Portocarero (EGP) occupational class schema. The multiple mediation tests are divided into three steps based on the following research questions. (1) Does occupational assortative mating affect short-term reproduction plans through the wife’s relative housework burden? (2) Does occupational assortative mating affect reproduction plans through the wife’s satisfaction with the housework division? (3) Does occupational assortative mating affect reproduction plans through the relationship between the wife’s relative burden of household chores and her satisfaction with that division?

## Literature review and research hypotheses

This study examines whether wives’ relative housework burden and satisfaction with their husbands’ contribution to housework can explain the heterogeneity in fertility plans based on occupational class mate selection preferences. The identified total-effect path is: occupational assortative mating patterns → plans for childbearing for the next 2 years. This study builds on existing research that confirms the presence of the total-effect model and seeks to fill the gaps by exploring the processes inherent in these tendencies. Statistically, the significance of the total effect is not necessary for testing or claiming indirect effects (Agler and Boeck, 2017; Preacher and Hayes, 2008; MacKinnon et al., 2000).

From a theoretical perspective, it is important to consider variations within occupational classes when analyzing fertility intentions. Prior research shows that occupational class is strongly associated with work–family compatibility, gender-role norms, and access to institutional support, all of which shape fertility decisions in distinct ways (Baizan, 2021; Kreyenfeld et al., 2023). Higher-status occupations typically provide greater job autonomy, shorter or more flexible work hours, and stronger social protection, enabling more egalitarian household arrangements and reducing the perceived cost of childbearing. In contrast, lower-status occupations are characterized by rigid schedules, limited family-friendly policies, and women in these jobs are more likely to encounter traditional gender-role expectations that assign primary household responsibilities to them. In this study, we distinguish three groups within the occupational hierarchy, as detailed in the Methods section.

The following sections review the theoretical rationale for the mediating roles of household labor division, wives’ satisfaction with housework, and their joint mediated effect.

### Relative household burden of wives

#### The effect of assortative mating on household division of labor

Resource bargaining theory (social exchange theory) is critical to understanding the social exchange of “who does more” and “who does less” in the distribution of household labor (Blood and Wolfe, 1960; Roman and Vogler, 1999). Differences in the socio-economic status of the husband and wife can help predict how household chores are divided. When the economic contributions of the two parties are nearly equal, or when the wife earns more than half of the income, the husbands tend to take on more housework, leading to a fairer distribution of chores (Du et al., 2020; Lam et al., 2012; Magda et al., 2024; Niu, 2017, 2020; Stauder and Röhlke, 2022; Xu and Liu, 2003). The relative resource hypothesis is gender-neutral (Chatot et al., 2023), suggesting that changes in either spouse’s relative or absolute resources will affect the division of labor.

However, numerous studies (Hochschild and Machung, 2012; Sun, 2018; Syrda, 2022) contradict resource theory. Evidence shows that when wives’ economic contributions exceed those of their husbands, their time spent in household labor actually increases, reducing their husbands’ housework participation. The relative resource hypothesis has been criticized for neglecting the social construction of gender inequalities. Consequently, scholars have adopted the “gender display” or “gender deviation neutralization” approach (Evertsson and Nermo, 2004, 2007; Liu et al., 2015; Sun, 2021; Tong and Liu, 2015; Yu and Xie, 2011; Zuo, 2002).

Essentially, gender perspectives depict the family as a “gender factory” (Fenstermaker, 2002), producing and reinforcing unequal positions between sexes in the domestic sphere. This imbalanced distribution, characterized by wives doing more or all of the housework, aligns with the Chinese ideal of a “virtuous wife and caring mother” (*xian qi liang mu*) and submissive femininity. In contrast, husbands do less or refuse to do housework to maintain dominance. Hypogamous marriages with female breadwinners conflict with traditional gender roles, where “men are the breadwinners and women are responsible for homemaking” (*nan zhu wai, nv zhu nei*). To compensate for deviating from these stereotypical “gendered resources” (Tichenor, 1999; Zuo and Bian, 2001), high-earning wives often engage in “doing gender” (West and Zimmerman, 1987), striving to adhere to or appear aligned with traditional roles as homemakers and caregivers.

Although “resource theory” and “gender theory” differ in explaining the relationship between a wife’s relative socio-economic status and her share of household chores, both suggest that marital pairing patterns may predict the wife’s relative housework burden. Additionally, gender theory may link resource theory to childbearing. According to Becker’s “New Home Economics” (Becker, 1985; Becker, 1991), a strict division of labor between paid labor market participants and unpaid family carers benefits higher fertility levels. However, with efforts to improve women’s economic empowerment, the female labor force has become an indispensable “half the sky” (*ban bian tian*) in socio-economic development. Tensions caused by the “dual burden” inevitably reduce the fertility intentions of career women (Anderson and Kohler, 2015; Goldin, 2021; Ji and Zheng, 2018; Wilkins, 2019; Yang, 2014). Therefore, reconciling “career-family incompatibility” by increasing husbands’ participation in household chores may have implications on fertility rates.

#### The impact of household division of labor on fertility

Two mainstream theories interpret the relationship between equality in the household labor division and fertility rates: the “Gender Revolution Theory” (Goldscheider et al., 2015) and the “Multiple Equilibrium Theory” (Connolly et al., 2016; Esping-Andersen et al., 2013; Esping-Andersen and Billari, 2015).

The Gender Revolution Theory argues that a fertility rebound relies on achieving gender equality in the domestic sphere, where husbands take on more household responsibilities, alleviating the “double burden” on wives (Goldscheider et al., 2015). The Multiple Equilibrium Theory suggests a U-shaped relationship between gender equality and fertility rates (Esping-Andersen and Billari, 2015). Fertility rates drop to their lowest when traditional gender roles are challenged, but societal institutions and couple-level interactions have not yet adapted to these changes. However, with the universalization of gender equality and the introduction of policies that reconcile career-family conflicts, equal sharing of household duties can be fulfilled, resulting in a stable increase in fertility rates (Esping-Andersen and Billari, 2015).

Ultimately, both theories converge on the view that increasing husbands’ commitment to domestic labor may facilitate multiple-child intentions or behaviors. However, the relationship between the division of household labor, women’s domestic involvement, and fertility is not uniform (Neyer et al., 2013; Zhou and Kan, 2019; Suero, 2023). A systematic review by Raybould and Sear (2021) shows that gender equality in domestic labor may be positively, negatively, or not at all related to fertility intentions and behaviors, depending on couples’ parity status and broader normative environments. Recent evidence from Italy further supports this parity-specific pattern: among childless working women, a heavier relative domestic workload is associated with lower intentions to have a child in the next 3 years, whereas among women who already have one child, women’s domestic burden is positively correlated with fertility intentions (García-Pereiro et al., 2025).

For instance, in Turkey, the unequal burden of household and childcare duties on women significantly contributes to declining fertility rates. Conversely, outsourcing chores, getting help, or equitable sharing of household labor with husbands can enhance women’s willingness to have more children (Kavas, 2019). In China, Yang (2022) suggests that when husbands participate in household chores, wives can dedicate more time and energy to paid work, which might lower their fertility desire due to career ambitions.

The relationship between equality in housework sharing and fertility behavior in America also exhibits a “U-shaped” pattern. Couples with the most traditional and egalitarian division of household labor are more likely to have a second child (Torr and Short, 2004). However, Schober’s (2013) study of British society presents a different view. They found that both husbands doing more/equally sharing chores and wives doing more/equally sharing chores (over 63% of the average chores) negatively impact the likelihood of entering parenthood, showing an “inverted U-shaped” relationship. Interestingly, the amount of housework each partner does may have inconsistent effects on family fertility. In Finland, a woman’s time spent on household chores negatively predicts subsequent fertility, while her husband’s involvement in household chores has a negligible effect on the number of offspring (Miettinen et al., 2015). Similarly, studies in mainland China (Yang, 2017) and Taiwan (Cheng and Hsu, 2020) found that the proportion of household labor undertaken by husbands has little effect on the likelihood of wives having a second child.

An exploratory study by Suero (2023) on women’s second-child intentions in Spain found an “inverted U-shaped” relationship between household labor division and fertility intentions. However, this trend was only evident in highly-educated women, while lower-educated women’s fertility intentions remained unaffected by their husbands’ participation in household chores. Suero (2023) speculates that this may be because highly educated women have greater bargaining power in marital relationships, leading them to place more importance on their husband’s housework contributions when engaging in fertility plans.

As women’s educational levels rise, there has been an increase in the proportion of educated women marrying less-educated men. According to van Bavel (2012), a more equal distribution of household labor can stimulate fertility. Therefore, the amount of housework a wife does may help explain how a woman’s choice of partner affects her reproductive plans. Hence, this study then proposes the following hypothesis:

*H1:* Occupational mating patterns influence reproduction plans through wives’ relative housework burdens.

### Wives’ satisfaction with the domestic division of labor

#### The effects of within-couple distribution of resources on satisfaction with housework

Concerning gender theory, the unequal division of household chores results from social construction, associating housework with femininity per traditional gender norms (Bianchi et al., 2012; Liu, 2022; Simulja et al., 2014). If couples strictly adhere to traditional gender roles, wives’ near-monopoly on household chores or husbands’ unwillingness to share them can seem reasonable rather than unfair (Carriero and Todesco, 2017; Cheung and Kim, 2018; DeMaris and Longmore, 1996). In such scenarios, wives do not perceive the unequal division of labor as unfair as long as it aligns with their role expectations of being “virtuous wives and caring mothers,” reflecting a “paradoxical contentment” (Major, 1993).

Women’s relative income and education levels influence not only their gender ideology (traditional or egalitarian) but also their ability to alleviate the burden of household chores, such as by procuring domestic services (Carriero and Todesco, 2018; Coltrane, 2000; Deole and Zeydanli, 2021; Kolpashnikova and Koike, 2021; Magda et al., 2024; Shu, 2004). Differences in gender ideology result in contrasting subjective standards of fairness (Flèche et al., 2020; Lennon and Rosenfield, 1994). Women’s socio-economic resources and their perceptions of fairness in the division of household chores support social exchange theory: women with lower salaries, education, or employment opportunities who rely on marriage to maintain their living standards lack the leverage to question inequality and are more likely to perceive an unequal division as fair (Lennon and Rosenfield, 1994).

#### Effect of satisfaction with household division of labor on fertility

Some scholars (Neyer et al., 2013) argue that the lack of consensus in studies exploring the impact of equal household chores division on fertility behaviors may stem from varying conceptualizations of “gender equality.” Neyer et al. (2013) suggest that the (un)equal distribution of unpaid care and domestic work between couples is only half the problem. Researchers should also consider how individuals feel about their respective arrangements. Only when both members of a couple perceive unfair household divisions as gender inequality can we expect a suppressive effect on childbearing to occur (*ibid.*). To fully explain variations in fertility behaviors, both the objective “equality” of labor division and the subjective perception of “equity” by women must be considered (Köppen and Trappe, 2019).

Studies examining the relationship between satisfaction with household tasks and fertility intentions (Cheung, 2023; Riederer et al., 2019; Snopkowski, 2023; Zhang et al., 2023) often use the wife’s subjective feelings toward housework sharing or her husband’s contribution as the link between her actual share of the housework and her fertility intentions. Zhang et al. (2023) found that in China, a husband’s greater participation in household tasks reduces the wife’s domestic burden and increases her satisfaction, leading to higher fertility intentions.

However, Zhang et al. (2023) overlooked the role of relative disparities in spousal socio-economic resources in determining the division of domestic work. Women evaluate domestic arrangements against context-specific standards of fairness shaped by their position within the marriage (Baxter, 2000). In occupationally homogamous unions, where spouses’ socioeconomic resources are relatively balanced, may hold stronger expectations for reciprocal domestic contributions (González et al., 2018), which raises the threshold for feeling satisfied with household divisions, even when the objective allocation is relatively equal.

By contrast, in occupationally asymmetric marriages, wives’ perceptions of fairness may diverge more markedly from objective domestic arrangements. Women married to higher-status husbands may normalize a heavier domestic burden and report relatively high satisfaction when their husbands meet minimal expectations (Lennon and Rosenfield, 1994). In hypogamous unions, housework arrangements may be evaluated against different normative benchmarks of fairness, as gender norms continue to define domestic labor as a key domain for enacting femininity (West and Zimmerman, 1987; Tichenor, 1999). In this sense, occupational mating patterns shape not only the division of labor itself, but also the interpretive frameworks through which wives evaluate it.

Accordingly, we propose the following hypothesis:

*H2:* Occupational mating patterns shape the wife’s satisfaction with housework division through her relative household burden, which in turn affects her short-term plans for an additional child.

As mentioned, “fairness” is subjective and does not necessarily mean that, objectively, the housework undertaken by each spouse is distributed equally. Some studies have analyzed fertility through the subjective lens of “fairness.” They suggest that women’s satisfaction with the division of labor or perceived fairness in their marital life has a stronger predictive power on their fertility motivations than the actual gendered division of labor (Dommermuth et al., 2017; Goldscheider et al., 2013; Luppi, 2016; Modiri and Sadeghi, 2021; Riederer et al., 2019; Torr and Short, 2004).

Moreover, the association between the division of household chores and wives’ satisfaction with it is not strong. In other words, the two mediating variables may not interact, indicating a parallel multiple-mediator model. Therefore, we propose an independent hypothesis for the intervening variable of housework satisfaction:

*H3:* Occupational mating patterns affect reproduction plans through wives’ satisfaction with housework arrangements.

## Methods

This study uses the 2020 dataset from the China Family Panel Studies (CFPS). This large-scale micro-survey adopts an implicit stratified, multi-stage, and probability-proportional-to-size sampling technique that integrates urban and rural areas (Xie et al., 2014). It covers 31 provinces/municipalities/autonomous regions in China, ensuring national and provincial representation. CFPS focuses on marriage and fertility behavior. A key advantage of CFPS is its comprehensive survey of all family members, providing data on each spouse’s future reproduction intentions, time spent on housework, satisfaction with their partner’s housework contributions, occupational class codes, and individual income from the previous year.

### Variables and sample characteristics

Previous survey questionnaires ascertained the ideal number of children from respondents; however, the 2020 respondents were asked whether they planned to have children in the next 2 years. An affirmative answer indicates strong fertility intention (Zheng, 2011), which is considered “one step away” from actualizing fertility plans (Miller and Pasta, 1995). This is reflective of the survey’s practical value for fertility prediction. Prior research suggests that short-term intentions, typically defined as childbearing plans within a time horizon of fewer than 3 years, are more concrete and more closely linked to behavioral implementation than long-term or abstract intentions (Philipov and Bernardi, 2012; Raybould and Sear, 2021). In this way, short-term intentions are formulated under relatively stable contextual constraints, they are less sensitive to unobserved life-course changes and therefore constitute a more appropriate outcome measure for cross-sectional analyses.

This survey question applied only to respondents under 50 years old who were married or cohabitating. As women bear the burden of pregnancy, this study focuses on the wife’s fertility expectation, treating it as a dichotomous variable. A value of “1” indicates the wife’s desire for a child within the next 2 years. CFPS2020 data was collected from July to December 2020, before the “three-child” policy was announced in May 2021.^1^ Considering the disincentivizing effect of the “over-birth” penalty policy, the sample is limited to families without a second child.^2^ The final valid sample includes 2,315 couples.

The key independent variable is the couple’s occupational class matching.^3^ Using the EGP class scheme (Erikson and Goldthorpe, 1992; Goldthorpe, 2007), this study converts the ISCO-88 occupational codes of husbands and wives from the CFPS2020 database into EGP class categories. The original 11 major EGP class categories are simplified. We define the higher I and II strata (managerial and professional technical personnel in party, enterprise, and public institutions) as the “core middle class,” the lower V to VIIb levels (workers and farmers) as the “ordinary laborers,” and the intermediate IVa to IIIb levels (small employers, self-employed, office staff, and commercial service personnel) as the “non-core middle class.^4^” We then compare the occupational classes of the couple. If they are complementary, their mating pattern is considered a “homogeneous marriage.” “Heterogeneous marriages” can be distinguished as “hypergamy (women marrying up)” and “hypogamy (women marrying down).”

The two mediating variables are the wife’s relative burden of housework and her satisfaction with her husband’s contribution to the housework. The “wife’s relative burden of housework” variable is calculated as the wife’s housework time/(wife’s housework time + husband’s housework time). A higher proportion indicates a heavier housework burden on the wife and less involvement from the husband, reflecting an unequal division of labor. Regarding the second mediator, a 5-point Likert scale measures the wife’s satisfaction with her husband’s contribution, from 1 (very dissatisfied) to 5 (very satisfied). We convert this to a binary variable reflecting the wife’s satisfaction with her husband’s housework contribution: scores of 1–3 are “0” (dissatisfied/neutral) and scores of 4–5 are “1” (satisfied).

This study also controls for factors influencing the division of housework and the wife’s fertility intentions or behaviors. These include the respondent’s age, gender, current urban residence, geographical region (Eastern, Western, and Central), parental or in-law assistance with housework and childcare, the logarithm of household income, the wife’s degree of economic dependence on her husband, and the wife’s belief or lack thereof that men should share half of the housework burden. According to previous literature (Brines, 1994; Greenstein, 2000; Liu, 2022; Sun, 2018), the wife’s economic dependency on her husband is calculated as: (wife’s previous year’s income – husband’s previous year’s income)/(wife’s previous year’s income + husband’s previous year’s income). This ratio ranges −1 to +1, indicating the transition from the complete economic dependency of the wife on her husband to the wife’s complete financial support of her husband. Considering that the division of housework—“who does more, who does less”—and the wife’s internalized feelings about her husband’s involvement may be a gendered performance adhering to the script of “who should do more, who should do less,” this study controls for the wife’s attitude toward the housework arrangement. Agreement with the statement “men should take on half of the housework” is rated on a 1–5 scale from “strongly disagree” (1) to “strongly agree” (5). Other control variables are included as numeric or “0/1” dichotomous variables.

Table 1 shows that 66.31% of the sample were urban dwellers, suggesting a high likelihood of rural residents having two children. Nearly 19.22% of respondents planned to have another child within 2 years. Respondents married someone with a similar EGP status (51.24%), and those indifferent “marrying within their class” (48.76%) were roughly equal. More women “married down” (31.27%) than “married up” (17.49%). This is likely because women predominated in routine non-manual and commercial service jobs (non-core middle class), whereas men made up the majority of working-class jobs (classified as ordinary laborers), giving women slightly higher occupational status.

**Table 1:** Descriptive statistics for the selected variables.

Variables	*N*	Mean (SD)/%
Whether plan to have children in the next 2 years		
No	1,870	80.78%
Yes	445	19.22%
Wife’s occupational class		
Core middle class	781	33.25%
Non-core middle class	722	30.74%
Ordinary laborers	846	36.02%
Husband’s occupational class		
Core middle class	721	28.77%
Non-core middle class	423	16.88%
Ordinary laborers	1,362	54.35%
Occupational assortative mating		
Hypergamy	405	17.49%
Core middle-class homogamy	374	16.16%
Non-core middle-class homogamy	177	7.65%
Ordinary laborers homogamy	635	27.43%
Hypogamy	724	31.27%
Wife’s relative housework burden	2,315	0.63 (0.25)
Whether the wife is satisfied with housework division		
No	990	42.76%
Yes	1,325	57.24%
Age	2,315	37.14 (8.04)
Gender		
Female	1,160	50.11%
Male	1,155	49.89%
Whether currently live in urban		
No	780	33.69%
Yes	1,535	66.31%
Geographical regions		
Eastern	1,125	48.60%
Central	639	27.60%
Western	551	23.80%
Whether parents or parents-in-law help with housework and childcare		
No	87	3.76%
Yes	2,228	96.24%
Log of household income	2,315	11.65 (0.77)
Wife’s economic dependency on her husband	2,315	−0.22 (0.52)
Whether the wife believes an equal share of the housework	2,315	4.27 (0.92)

The mean share of housework for wives was 0.63, indicating that they were the main contributors. Despite this, 57.24% of wives were satisfied with their housework arrangements. This suggests that women’s subjective evaluations of housework division may not align with the objective distribution of time invested. Almost all families (96.24%) had help from older generations with chores and childcare. The average degree of wives’ economic dependency on their husbands was −0.22, indicating that most women remained economically dependent. The sample reflects the traditional gender stereotype of the male breadwinners and female housekeepers. The mean level of wives’ agreement with gender equality in household chores is 4.27, between “agree” and “strongly agree,” which does not align with the actual division of labor.

Table 2 presents the distribution of wives’ short-term fertility intentions across different occupational assortative mating patterns. Clear heterogeneity emerges across mating groups. Wives in core middle-class homogamous marriages exhibit the highest proportion of plans to have a child within the next 2 years, whereas fertility intentions are weakest among ordinary laborer homogamous couples. Among heterogeneous marriages, wives who marry down display slightly higher fertility intentions than those who marry up, and both heterogeneous groups report higher fertility intentions than non-core middle-class homogamous and ordinary laborer homogamous marriages.

**Table 2:** Distribution of wives’ short-term fertility intentions by occupational assortative mating patterns.

Occupational assortative mating	Whether plan to have children in the next 2 years		Total
	No	Yes	
Hypergamy	328 (80.99%)	77 (19.01%)	405 (100%)
Core middle-class homogamy	269 (71.93%)	105 (28.07%)	374 (100%)
Non-core middle-class homogamy	144 (81.36%)	33 (18.64%)	177 (100%)
Ordinary laborers homogamy	553 (87.09%)	82 (12.91%)	635 (100%)
Hypogamy	576 (79.56%)	148 (20.44%)	724 (100%)
Total	1,870 (80.78%)	445 (19.22%)	2,315 (100%)

These descriptive patterns suggest that occupational assortative mating is systematically associated with women’s fertility intentions, underscoring the importance of examining the mechanisms through which different mating patterns shape fertility plans. The subsequent multivariate and mediation analyses build on this descriptive evidence to assess whether differences in household labor division and wives’ satisfaction help explain this observed heterogeneity.

### Analytical strategy

This study uses GSEM to explore the mediating effects of wives’ housework burden and satisfaction with their husbands’ housework contribution. SEM/GSEM is recommended by scholars (e.g., Fang et al., 2014; Gunzler et al., 2013) as the most suitable model for analyzing and verifying multiple mediation effects, allowing for simultaneous testing of multiple indirect and direct effects. The response variables include both numerical and dichotomous variables; hence, GSEM is preferred, with the equations as follows:


$$\begin{array}{l} \text{Housework}\_\text{burden}=a_{k-1}\text{Occupational}\_\\ \text{class}\_\text{matc}h_{k-1}+\gamma_{1}\text{Control}\_\text{variables}+e_{1} \end{array}$$ (1)

$$\begin{array}{l} \text{Wives}\_\text{satisfaction}\_\text{with}\_\text{housework}=e_{k-1}\text{Housework}\_\\ \text{burde}n_{k-1}+c_{k-1}\text{Occupational}\_\text{class}\_\text{matc}h_{k-1}\\ +\gamma_{2}\text{Control}\_\text{variables}+e_{2} \end{array}$$ (2)

$$\begin{array}{l} \mathrm{Pr}(\text{Have}\_\text{another}\_\text{child})=c_{k-1}^{\prime }\text{ccupational}\_\text{class}\_\\ \text{matc}h_{k-1}+b_{p-1}\text{Housework}\_\text{burde}n_{p-1}+d_{p-1}\text{Wives}\_\\ \text{satisfaction}\_\text{with}\_\text{housewor}k_{p-1}+\gamma_{3}\text{Control}\_\text{variables}+e_{3} \end{array}$$ (3)

**Figure 1:**
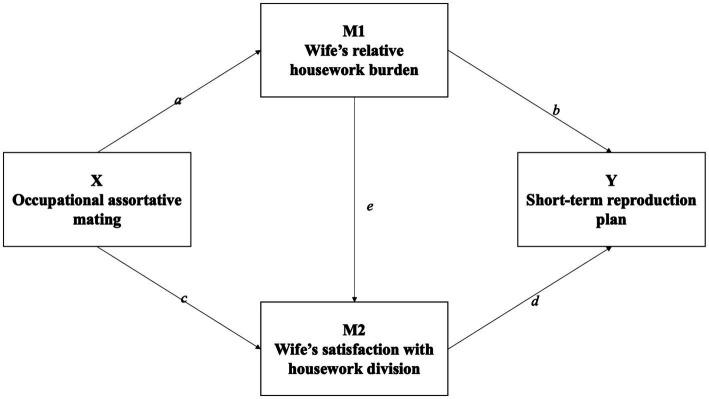
Decomposition of the total effect.

Depending on the response variables, Equations 1, 2 used multiple linear regression, while Equation 3 used a binary Logit model. The control variables were consistent across all equations. As illustrated in Figure 1, the three equations cover four paths: Equation 1 estimates the effect of occupational assortative mating on the wife’s relative housework burden (X → M1); Equation 2 calculates how the wife’s housework participation affects her satisfaction with housework (M1 → M2), and estimates the effect of occupational matching on satisfaction while controlling for housework participation (X → M2); Equation 3 estimates the effect of occupational matching on fertility plans after controlling for the two mediating variables (X → Y).

In Figure 1, the indirect effect of M1 is the product of $a_{k-1}$ and $b_{p-1}$ , and the indirect effect of M2 is $c_{k-1}\times d_{p-1}$ . The effect of M1 on M2 is $a_{k-1}\times e_{k-1}\times d_{p-1}$ . The total indirect effect is the sum of these three. The significance of these indirect effects is tested using the bias-corrected non-parametric percentile Bootstrap method, which captures data variability and complexity. We re-sampled 1,000 times and set a 95% confidence interval.

## Results

Table 3^5^ shows the results of the three equations in the GSEM model. We focus on the coefficients and significance of *a*, *b*, *c*, *d*, and *e* shown in Figure 1, which are crucial for calculating and validating indirect effects.

**Table 3:** Path coefficients of the GSEM model.

	Model 1	Model 2	Model 3
	Wife’s relative housework burden	Wife’s satisfaction with housework	Fertility plan in two years
	Coefficients (SE)	Coefficients (SE)	Coefficients (SE)
Occupational assortative mating (reference: core middle-class homogamy)			
Hypergamy	0.060*** (0.016)	0.249* (0.135)	−0.473** (0.198)
Non-core middle-class homogamy	0.064** (0.012)	0.218 (0.168)	−0.903** (0.263)
Ordinary laborers homogamy	0.048** (0.016)	0.142 (0.136)	−0.791*** (0.217)
Hypogamy	0.026* (0.015)	0.415** (0.125)	−0.774*** (0.175)
Wife’s relative housework burden		−1.023*** (0.151)	0.526** (0.255)
Whether the wife is satisfied with housework division (reference: no)			
Yes			0.2490** (0.1223)
Control variables	*Controlled*	*Controlled*	*Controlled*
*N*	3,389	3,381	2,315
AIC			6,284.999
BIC			6,566.901

*p ≤ 0.1, **p ≤ 0.5, ***p ≤ 0.001.

In Model 1, compared to the homogamous core middle class, all other mating patterns have significantly positive coefficients, indicating that wives in core middle-class homogamous marriages experienced the lowest housework burden. Wives in non-core middle-class homogamy bore the heaviest burden, followed by those with lower occupational classes than their husbands. Model 2 shows a negative correlation between wives’ relative burden of housework and their satisfaction with the arrangements. As the wife’s share of household chores increased, her dissatisfaction with her husband’s housework contribution grew. Satisfaction varied across occupational mating patterns. The coefficients for heterogamy are significant and positive, indicating that compared to core middle-class homogamy, wives in both “marrying up” and “marrying down” marriages were more likely to be satisfied with their husbands’ contributions. Wives experienced greater housework satisfaction in hypogamous marriages. Combining results from Model 1 and Model 2 in core middle-class homogamy shows that a lighter domestic burden on wives did not lead to higher satisfaction with husbands’ involvement. However, in hypergamy, a heavier housework burden did not necessarily reduce wives’ housework satisfaction.

Model 3 reveals a trend that contradicts the gender revolution theory. Women with heavier housework burdens tend to report higher short-term fertility intentions. Wife’s housework satisfaction is positively associated with (re)fertility intentions. Negative coefficients for other mating patterns relative to the intraclass marriage of the core middle class suggest that core middle-class families had the strongest intention to have another child. Non-core middle-class couples had the lowest intention. Ordinary laborers homogamy was the second lowest. Compared to core middle-class homogamy, fertility intentions in heterogamous marriages are higher than in other homogamous types. Motivation to have additional child is stronger in women “marrying up” than those “marrying down.”

Table 4 shows the indirect effect sizes and BC-corrected 95% confidence intervals adjusted for each path of the two mediating variables. An indirect effect is confirmed if the confidence interval does not contain 0. As the independent variable X is multi-categorical, we compare the relative indirect effects of the wife’s relative housework burden (M1) and the wife’s satisfaction with the division of housework (M2) on the relationship between occupational mating patterns and fertility intentions against the core middle-class homogamous marriages (Fang et al., 2017; Hayes and Preacher, 2014).

**Table 4:** The mediation effect-sizes and confidence intervals of M1 and M2 for a multi-categorical X.

	M1	M2	M1 → M2	M1–M2	Total indirect effect
Hypergamy	**0.031 [0.003, 0.077]**	**0.06 [0.005, 0.227]**	**−0.015 [−0.039, −0.002]**	−0.031 [−0.186, 0.036]	**0.0782 [0.013, 0.227]**
Non-core middle-class homogamy	**0.034 [0.004, 0.085]**	0.054 [−0.013, 0.211]	**−0.016 [−0.043, −0.002]**	−0.021 [−0.181, 0.061]	0.072 [−0.006, 0.214]
Ordinary laborers homogamy	**0.025 [0.003, 0.064]**	0.035 [−0.017, 0.167]	**−0.012 [−0.035, −0.001]**	−0.011 [−0.140, 0.052]	0.049 [−0.013, 0.165]
Hypogamy	**0.014 [0.000, 0.046]**	**0.103 [0.015, 0.284]**	**−0.007 [−0.022, −0.000]**	−0.089 [−0.272, 0.002]	**0.111 [0.020, 0.276]**

The bold values represent statistically significant results.

Wives’ relative housework burden has a significant indirect effect on fertility intentions across all mating types. A higher share of household labor for wives increases the likelihood of expecting a child within 2 years (Model 3). Compared to core middle-class homogamy, wives in all other mating types bore a heavier domestic burden, leading to positive relative indirect effects through M1. Therefore, H1 was confirmed for all pairing groups. However, Model 3 shows negative direct effects for these mating types. Significant direct and indirect effects with opposite signs indicate a suppression effect (Demming et al., 2017; MacKinnon et al., 2000; Preacher and Hayes, 2008; Wen and Ye, 2014). More specifically, wives’ relative housework burden partially counterbalances the negative association between occupational assortative mating and fertility intentions.

For occupational hypergamy, wives’ relative domestic burden suppresses 6.6% ( $|\frac{0.031\;}{-0.473}|$ ) of the negative direct effect. The negative impact of non-core middle-class homogamous marriage on fertility intentions is offset by 3.8% ( $|\frac{0.034}{-0.903}|$ ) due to their household labor division. For ordinary laborer homogamy, 3.2% ( $|\frac{0.025}{-0.790}|$ ) of the negative direct effect is suppressed. Hypogamy’s suppression effect on fertility intentions is 1.8% ( $|\frac{0.014}{-0.774}|$ ). Although these suppression effects are quantitatively modest, they are theoretically meaningful, suggesting that household labor division can partially buffer some of the disadvantages associated with certain occupational mating patterns.

Wives in both types of occupational heterogamy reported higher satisfaction with their husbands’ housework contributions than in core middle-class homogamy (Model 2). This satisfaction positively predicts their fertility intentions (Model 3). Therefore, the indirect effects of M2 are positive, contrasting with the direct effects (Model 3), indicating suppression effects. These suppression effects in the relationship between women occupational heterogamy and fertility intentions are 13.1% ( $|\frac{0.062}{-0.473}|$ ) and 13.3% ( $|\frac{0.103}{-0.774}|$ ), respectively. Negative associations between M1 and M2 weaken either model’s positive (indirect) predictive effect on the expectations of having a second child, revealing multiple suppression effects. Therefore, both H2 and H3 were verified in heterogamous pairings only.

The serial indirect effects of M1 → M2 are smaller than the specific indirect effects of M1 or M2, yet the total indirect effect of all three respective paths remains positive. In both types of heterogamous marriages, the differences in indirect effects of M1 and M2 are not statistically significant, making it difficult to determine which has a stronger suppression effect.

This study aimed to test the mediating effects of objective and subjective aspects of housework division on fertility plans. The wife’s share of household labor was initially treated as a numerical variable to demonstrate the probability change from no fertility plan to having one as the wife’s relative domestic burden increased. However, this approach did not capture the non-linear trend between actual household labor division and fertility intentions, which related studies suggest follows a “U-shape” (Torr and Short, 2004) or “inverted U-shape” (Schober, 2013; Suero, 2023) pattern. Following Suero’s (2023) study, we recorded the wife’s household labor share variable into four intervals: “0–40%,” “41–60%,” “61–80%,” and “≥81,” and refitted Model 3. Consistent with Model 2, Model 4 (Table 5) showed that the lower the share of household chores the wife undertook, the more satisfied she was with her husband’s household contributions, with the highest satisfaction when the husband did most of the housework.

**Table 5:** The non-linear relationship between the wife’s relative housework burden and wives’ fertility intentions.

	Model 4	Model 5
	Wife’s satisfaction with housework	Fertility plan in two years
	Coefficients (SE)	Coefficients (SE)
Occupational assortative mating (reference: Core middle-class homogamy)		
Hypergamy	0.228* (0.134)	−0.432** (0.196)
Non-core middle-class homogamy	0.210 (0.167)	−0.817** (0.262)
Ordinary laborers homogamy	0.142 (0.135)	−0.731** (0.216)
Hypogamy	0.380** (0.124)	−0.710*** (0.174)
Wife’s relative housework burden (reference: ≥81%)		
0–40%	0.703*** (0.111)	−0.362* (0.186)
41–60%	0.524*** (0.099)	−0.102 (0.168)
61–80%	0.463*** (0.093)	−0.195 (0.160)
Whether the wife is satisfied with housework division (reference: no)		
Yes		0.271** (0.122)
Control variables	*Controlled*	*Controlled*
*N*	3,436	2,348
AIC	4,485.013	1,795.472
BIC	4,589.428	1,899.176

*p ≤ 0.1, **p ≤ 0.5, ***p ≤ 0.001.

As anticipated, Model 5 indicates a non-linear, “N-shaped” relationship between the wife’s relative domestic burden and her fertility intentions. Compared to wives performing ≥81% of housework, only those in the 0–40% group where husbands performed the majority of domestic labor showed significantly lower fertility intentions within 2 years. Coefficients for the 40–60% and 61–80% groups were not statistically significant, though the pattern suggests that fertility intentions tend to be relatively higher when housework is either highly unequal (wife does most) or moderately shared.

## Discussion and conclusion

The unequal division of labor among career women within family-oriented institutions is a fundamental driver of the low fertility and negative population momentum in many countries (McDonald, 2000a, 2000b). Research on the relationship between mating patterns and fertility intentions remains largely limited to verifying correlations without explaining how a spouse’s identity affects the number of children they plan to have. Meanwhile, the impact of subjective and objective aspects of household division of labor on fertility is seldom discussed in Chinese society (Cheung, 2023; Zhang et al., 2023). This study examines how couples’ relative resources shape the actual division of household chores and the wife’s subjective satisfaction with it. We integrate equality and equity theory to explain how occupational assortative mating affects the wife’s fertility intentions.

We found that traditional or equalitarian household division of labor increases the wife’s fertility intentions, aligning partly with the trend pointed out by the MEF theory (Esping-Andersen and Billari, 2015). When the gender division of labor is in a transitional phase from traditional to egalitarian, the balance between the idea of “male dominates outside, female dominates inside” breaks. Work-family role conflicts exhaust women, yet supportive government policies for work-family balance, such as flexible jobs for mothers and effective childcare, are lacking. This “policy lag” leads to declining fertility rates (McDonald, 2000a, 2000b). Contrary to the “U-shaped” relationship found in other studies between the proportion of housework borne by women and fertility behavior (Torr and Short, 2004), this study finds that a heavier housework load on husbands also reduces wives’ fertility motivations, creating an “N-shaped” relationship. As noted by Okun and Raz-Yurovich (2019), increased male housework contribution can create a “double burden,” simultaneously managing work and domestic responsibilities, which may suppress their fertility intentions, and in turn affects their wives’ fertility intentions. It should be noted that the 0–40% group may represent a highly selective set of women with lower fertility desire; for example, they may serve as the primary economic providers in the household and prioritize career development. Panel data would be necessary to examine whether women with lower housework burdens tend to have lower fertility intentions.

Occupational mating patterns influence the division of household chores between spouses. Wives in core middle-class families bear the lowest housework burden, while those “marrying up” face increased burdens. This suggests that “resource theory” (Blood and Wolfe, 1960) has some explanatory power. Notably, wives in hypogamous unions experience a lighter housework burden than those in hypergamous marriages, yet still shoulder more housework than their counterparts in core middle-class homogamous unions. This pattern cannot be fully accounted for by relative resource differentials alone. A plausible interpretation is that women in hypogamous marriages may engage in compensatory gender performance (Tichenor, 1999; Zuo and Bian, 2001), by voluntarily assuming greater domestic responsibility, in order to reaffirm normative gender expectations when their occupational status exceeds that of their husbands. Such a “doing gender” mechanism (West and Zimmerman, 1987) may therefore operate alongside resource-based dynamics, shaping housework allocation in occupationally asymmetric unions.

Additionally, “equity theory” (Köppen and Trappe, 2019; McDonald, 2000a, 2000b; Neyer et al., 2013) shows that a wife’s satisfaction with housework division affects her fertility intentions. The more equitable she perceives her share, the more likely she is to plan for more children. However, this study finds that women’s (dis)satisfaction does not always align with their actual share of chores. Their (dis)advantaged position relative to their husbands may explain these paradoxical patterns, where perceived satisfaction contrasts with the objective division of housework. In core middle-class couplings, wives contribute the least to housework but do not experience higher housework satisfaction. In “marrying up” marriages with a more unequal division of household chores, wives’ satisfaction with their husband’s contributions is not low. In hypogamous marriages, wives’ perceptions align with their actual housework shares, leading to more positive evaluations of her contributions. Therefore, this study refutes previous research on the consistency between objective “equality” in housework division and the subjective “equity” perceived by the wife (Cheung, 2023; Riederer et al., 2019; Snopkowski, 2023; Zhang et al., 2023).

Prior research (Cheung, 2023; Zhang et al., 2023) suggests that subjective perceptions of domestic labor link the relative housework burden and fertility intentions. This study agrees that both objective and subjective components of housework influence variations in the wife’s fertility expectations. After introducing intra-couple relative socio-economic resources as an independent variable reveals contradictions between actual housework allocation and wives’ satisfaction with the distribution, which were not fully addressed in previous studies. Mediation analysis shows that the subjective and objective components of domestic labor division affect the impact of wives’ relative occupational status on their short-term fertility plans. Overall, the mechanisms revealed align with our hypotheses: occupational mating patterns influence fertility intentions partly through wives’ relative housework burdens (H1), which in turn affect their satisfaction with household arrangements (H2), and also work through wives’ satisfaction as an additional, independent pathway (H3).

Compared to the core middle-class homogamy, all other occupational mating patterns negatively affect fertility intentions. For each mating type, the relative indirect effects of wives’ relative housework burden or their satisfaction with that distribution are statistically significant and positive, indicating the presence of suppression effects. Notably, multiple suppression effects only occur in the relationship between the women “marrying up” or “marrying down” heterogeneous marriages and their fertility intentions.

These heterogeneous patterns reflect the dual role of status asymmetry in such unions. In hypergamous marriages, gender-normative expectations tend to amplify women’s sense of fairness even when their domestic workload is heavy, because conforming to traditional gender roles is interpreted as legitimate compensation for their lower socioeconomic position. In contrast, in hypogamous marriages, women often engage in gender performance to counteract the symbolic tension created by violating the “male-breadwinner” norm, which may lead them to maintain or even overperform domestic work while simultaneously downplaying feelings of unfairness.

Such divergences between objective housework burden and their reported satisfaction create conditions under which multiple suppression effects are more likely to emerge: the indirect path through satisfaction can either reinforce or offset the path through actual burden because the two components move in different directions. By contrast, in non-core middle-class or ordinary worker homogeneous marriages, only the wives’ share of household chores significantly impacts fertility expectations, not their satisfaction with housework.^6^ A likely explanation is that in these unions, housework is structured primarily by material constraints and task-related demands; thus, women’s evaluations of fairness have limited independent influence once their actual workload is accounted for.

This study has some methodological limitations. First, this study uses couples’ current occupations, as reported in the CFPS2020, to measure occupational assortative mating. Ideally, this concept refers to occupational sorting prior to marriage; however, the CFPS does not collect retrospective data on pre-marital occupations, and only the 2020 wave includes questions about fertility intentions. While CFPS is a longitudinal survey, the number of respondents who were unmarried in earlier waves and later married by 2020 is extremely limited, making it difficult to construct a reliable subsample for analysis. As a result, we use current occupations as the best available proxy, acknowledging that this may conflate pre-marital sorting with post-marital occupational changes, particularly for women. When interpreting the results, this limitation suggests caution in attributing causality to occupational sorting per se. The estimated effects may partially reflect post-marital labor market adjustments rather than purely pre-marital matching preferences. Future research with more detailed occupational histories could help disentangle these dynamics more clearly.

Second, the ideal-typical female full-time homemaker–male breadwinner household is not fully captured in this study. This study includes only women who reported their current occupation in the CFPS survey, as occupational assortative mating is constructed using the EGP class schema, which relies on respondents’ current occupational information. As a result, a very small proportion of couples in which women identified themselves as full-time homemakers were inevitably excluded from the analytical sample. In contemporary China, dual-earner households are highly prevalent, and therefore the exclusion of homemakers is unlikely to substantially bias the main findings. Nevertheless, male-breadwinner households represent a theoretically important case of assortative mating, in which the gender division of housework is likely to be the most traditional. Future research should explicitly incorporate this group in order to better capture the full U-shaped relationship between gender equality and fertility proposed by both the Gender Revolution Theory and Multiple Equilibrium Theory.

Third, it does not account for the impact of husbands’ childcare time on wives’ intentions for a second child, despite evidence that this involvement influences fertility intentions more than equitable housework distribution (Cheng and Hsu, 2020; Miettinen et al., 2015). This is because the CFPS2020 questionnaire limits this data to respondents with children under 16, reducing the sample size^7^ and the credibility of the results. Forth, while the study measures “how much housework” the wife undertakes, it does not determine “what kinds of housework” are undertaken. Research discussing the gender segregation of housework (Altintas and Sullivan, 2017; Guppy and Luongo, 2015) shows that typically differentiated “feminine” tasks (e.g., laundry, cleaning, meal preparation) are notably more routine and time-consuming than non-routine, on-demand “masculine” tasks (e.g., appliance repairs, car maintenance). The impact of husbands’ involvement in stereotypically feminine tasks—as opposed to those enacting masculine identities—on wives’ fertility intentions warrants further exploration.

Lastly, cross-sectional data cannot control for endogeneity issues. The causal relationships between key variables require more robust evidence (e.g., using longitudinal data). Women desiring a second child may choose flexible, lower status jobs to fulfill “caregiving” roles. However, this study finds that wives in core middle-class homogamy have the strongest fertility intentions, while hypergamy does not support these intentions. Thus, the reverse causal relationship between women’s relative occupational status and fertility intentions is untenable. Additionally, when women choose to undertake childbearing and depend economically on their husbands or earn much less, their household chores increase. This is evidenced by the greater share of chores borne by women who “marry up.” Given the negative correlation between housework and satisfaction and the positive correlation between the wife’s satisfaction and her fertility intentions, a return to “traditional” roles may not motivate more children. Therefore, the study’s findings are unlikely to be driven by reverse or bidirectional causation.

## Data Availability

The original contributions presented in the study are included in the article/supplementary material, further inquiries can be directed to the corresponding author.

## Footnotes

1 “Decision of the central committee of the CPC and the state council on optimizing birth policies to promote long-term and balanced population development”. 2021. Central People’s Government of the PRC. Retrieved January 1, 2024. (https://www.gov.cn/zhengce/2021-07/20/content_5626190.htm).

2 This includes both childless families and families with only one child. Sub-group analyses were not conducted due to the small proportion of childless couples. The focus of this study is on short-term fertility intentions for a subsequent birth within 2 years, rather than on completed fertility by parity. In this sense, the dependent variable already conditions on parity: childless respondents are asked about the transition to first birth, while parents of one child are asked about the transition to second birth. For couples who have already had one child, household labor division and wives’ satisfaction may have already reached a relatively balanced state. This pre-existing condition implies that the observed association between household division, satisfaction, and fertility intentions may be influenced by a selection effect. To disentangle this and examine whether a more equitable division or higher satisfaction truly increases the likelihood of a second child, longitudinal panel data would be required.

3 This study focuses on occupational rather than educational assortative mating because occupation more directly captures individuals’ current economic conditions, job stability, and work–family constraints, which are closely related to household labor division and short-term fertility planning, whereas education mainly reflects stable human capital accumulated earlier in the life course.

4 In this study, the “core middle class” is defined as encompassing both the higher and lower service classes, which are characterized by relatively stable employment, higher educational attainment, greater income security, and stronger access to institutionalized welfare benefits. Members of this group typically occupy professional, managerial, or technical positions and enjoy comparatively higher social status and life chances. By contrast, the “non-core middle class” (also referred to as the marginal middle class) comprises occupational groups located at the boundary between the middle class and manual labor, such as routine clerical and low-level administrative workers. Although these occupations are formally classified as non-manual, they are marked by lower job autonomy, weaker employment security, and more limited opportunities for upward mobility.

5 Complete model estimates, including coefficients for all control variables, are reported in Appendix.

6 Significance levels should not be interpreted as definitive evidence for the presence or absence of a relationship, given their sensitivity to sample size, measurement error, and model specification. In this study, the “non-significant” indirect pathway is discussed only to indicate that its estimated magnitude is comparatively small under the current model, not to suggest that the mechanism is theoretically absent.

7 If including the variable measuring childcare time in the model, the sample size would reduce to 366 while keeping other variables constant.

## Appendix

### A1: Path coefficients of the GSEM model (full)

**Table:**

	Model 1	Model 2	Model 3
	Wife’s relative housework burden	Wife’s satisfaction with housework	Fertility plan in 2 years
	Coefficients (SE)	Coefficients (SE)	Coefficients (SE)
Occupational assortative mating (reference: core middle-class homogamy)			
Hypergamy	0.060*** (0.016)	0.249* (0.135)	−0.473** (0.198)
Non-core middle-class homogamy	0.064** (0.012)	0.218 (0.168)	−0.903** (0.263)
Ordinary laborers homogamy	0.048** (0.016)	0.142 (0.136)	−0.791*** (0.217)
Hypogamy	0.026* (0.015)	0.415** (0.125)	−0.774*** (0.175)
Wife’s relative housework burden		−1.023*** (0.151)	0.526** (0.255)
Whether the wife is satisfied with housework division (reference: no)			
Yes			0.2490** (0.1223)
Age	0.001 (0.000)	0.015*** (0.004)	−0.191*** (0.011)
Gender (reference: female)			
Male	−0.005 (0.008)	−0.050 (0.072)	0.249** (0.121)
Whether currently live in urban (reference: No)			
Yes	0.000 (0.010)	−0.214** (0.084)	0.052 (0.141)
Geographical regions (reference: Eastern)			
Central	0.013 (0.010)	0.033 (0.087)	−0.155 (0.152)
Western	−0.008 (0.011)	−0.249** (0.092)	0.482** (0.144)
Whether parents or parents-in-law help with housework and childcare (reference: No)			
Yes	−0.002 (0.014)	−0.484*** (0.123)	0.135 (0.405)
Log of household income	0.004 (0.006)	−0.033 (0.050)	−0.121 (0.091)
Wife’s economic dependency on her husband	−0.053*** (0.008)	−0.124* (0.070)	0.292** (0.117)
Whether the wife believes an equal share of the housework	−0.010** (0.005)	0.093** (0.039)	0.003 (0.068)
*N*	3,389	3,381	2,315
AIC			6,284.999
BIC			6,566.901

**p* ≤ 0.1, ***p* ≤ 0.5, ****p* ≤ 0.001.
